# The histology of rhynchosaur (Diapsida, Archosauromorpha) ankylothecodonty

**DOI:** 10.1111/joa.70037

**Published:** 2025-08-27

**Authors:** Gabriel Mestriner, Gregory F. Funston, Sterling J. Nesbitt, Júlio C. A. Marsola, David C. Evans, Christian A. Sidor, Max C. Langer, Aaron R. H. LeBlanc

**Affiliations:** ^1^ Departamento de Biologia Universidade de São Paulo Ribeirão Preto Brazil; ^2^ Department of Ecology and Evolutionary Biology University of Toronto Toronto Ontario Canada; ^3^ Department of Natural History Royal Ontario Museum Toronto Ontario Canada; ^4^ Department of Earth and Planetary Sciences University of California Davis Davis California USA; ^5^ Department of Anatomical Sciences Renaissance School of Medicine, Stony Brook University, Health Sciences Center New York USA; ^6^ Department of Geosciences Virginia Tech Blacksburg Virginia USA; ^7^ Universidade Tecnológica Federal do Paraná Paraná Brazil; ^8^ Department of Biology and Burke Museum University of Washington Seattle Washington USA; ^9^ Faculty of Dentistry, Oral & Craniofacial Sciences King's College London London UK

**Keywords:** alveolar bone, ankylosis, Ankylothecodont, dental histology, heterochrony, tooth attachment

## Abstract

The study of the connection between the teeth and the jaw is important for understanding the palaeobiology of vertebrates, but inconsistent terminology and incomplete sampling have made it difficult to assess the evolutionary significance of some of the related characters. Among archosauromorphs, tooth attachment in dinosaurs and crocodylians is nearly identical to that of mammals in featuring a ligamentous connection (gomphosis), whereas closely related forms appear to have teeth fused to the jaws (ankylosis), as in most other amniotes. Hence, studying tooth attachment of stem‐archosaurs is pivotal to characterize the main shifts in tooth attachment seen in the lineage. Here, we analyze the tooth attachment of rhynchosaurs — a group of quadrupedal herbivorous archosauromorphs that played a key role as primary consumers in many Triassic communities. Their dentition consists of multiple rows of marginal teeth with posterolingual addition of teeth during growth, but their tooth attachment has not been documented in a modern context. Histological data from three rhynchosaur specimens from the Middle Triassic Manda Beds of Tanzania show that, although ankylosed, rhynchosaur teeth are surrounded by an extensive network of Sharpey’s fibers, layers of cementum, and well‐defined zones of alveolar bone. What has been previously described as “spongy bone of attachment” in fact encompasses the same attachment tissues present in mammals, dinosaurs, and crocodylians, albeit completely mineralized in mature teeth. Analysis of different stages of tooth development shows that ankylosis occurs by the growth of alveolar bone towards the cellular cementum, which eventually mineralizes the soft ligament. This suggests that the tissues conflated as “bone of attachment”—alveolar bone, periodontal ligament, and cellular cementum—are homologous across Archosauromorpha. Our data add to a growing body of evidence that heterochronic changes to the timing and extents of mineralization, not convergent evolution to mammal‐like attachment tissues, led to the independent evolution of gomphosis across many amniote lineages, including archosauromorphs.

## Graphical abstract

Dental evolution is a major topic in vertebrate palaeontology and provides key insights into the evolutionary history of several amniote clades. This study investigates rhynchosaur ankylothecodonty within the broader evolutionary context of Archosauromorpha. Our results show that rhynchosaur dentition comprises the plesiomorphic amniote tissues — alveolar bone, cellular cementum, and the mineralized periodontal ligament — challenging assumptions about the so‐called “bone of attachment.” Within this framework, rhynchosaurs exhibit rapid ankylosis, adding to a growing body of evidence that heterochronic shifts in mineralization timing led to the evolution of gomphosis across many amniote clades, including mammals, dinosaurs, and crocodylians.

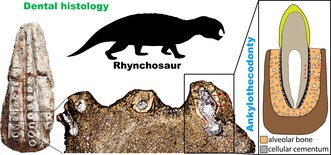

## 1 INTRODUCTION

The study of tooth attachment and implantation has yielded valuable insights into the palaeobiology of vertebrates. Whereas the term “tooth implantation” categorizes teeth by their spatial relation to the jaws, “tooth attachment” distinguishes teeth that are fused to the bone (ankylosis) from those suspended by a periodontal ligament (gomphosis). In the latter case, the presence of periodontal ligaments (a network of collagen fibers) has multiple functions, such as providing a flexible attachment for the tooth in the bony socket, which facilitates post‐eruptive tooth movement, and also acts as a sensory system to help in proper positioning of the jaws during mastication (Nanci, 2013). However, the evolutionary significance of tooth attachment has been difficult to assess due to historical conflation with tooth implantation terminology (Mestriner et al., 2025), resulting in misinterpretations of the reptilian attachment tissues (sometimes called “bone of attachment”) and their homology with those of mammals: root cementum, periodontal ligament, and alveolar bone (Caldwell, 2007; Caldwell et al., 2003; LeBlanc, 2023; LeBlanc et al., 2017, 2018, 2020; Maxwell, Caldwell, & Lamoureux, 2011a, 2011c; Maxwell, Caldwell, Lamoureux, & Budney, 2011; Mestriner et al., 2022; Palci et al., 2021).

Tooth attachment in dinosaurs and crocodylians is nearly identical to that of mammals (LeBlanc et al., 2017), but stem‐archosaurs appear to have ankylosed teeth (Mestriner et al., 2022), similar to most other amniotes (LeBlanc & Reisz, 2013). This casts doubt on a single origin of the gomphosis within Archosauria. Even though the three attachment tissues (cementum, periodontal ligament, and alveolar bone) are homologous in archosaurs regardless of tooth attachment mode (LeBlanc et al., 2017; Mestriner et al., 2022), it is uncertain when and how the ancestral ankylosed condition transformed into the persistent complex of periodontal ligaments found in dinosaurs and crocodylians (Mestriner et al., 2022). Hence, studying tooth attachment of stem‐archosaurs (non‐archosaurian archosauromorphs) is pivotal to identify the main shifts in tooth attachment seen in the lineage, and to understand the mechanisms through which tooth attachment changed across Archosauromorpha. Here, we analyze tooth attachment in rhynchosaurs (*Stenaulorhynchus stockleyi*), a group of quadrupedal herbivorous archosauromorphs that played a key role as primary consumers in many Triassic ecosystems. The group was first recorded shortly after the Permo‐Triassic mass extinction in the Early Triassic of South Africa (Ezcurra et al., 2014), diversified during the Middle Triassic, and became nearly cosmopolitan during the Late Triassic, with records in present‐day South and North America, continental Africa, Madagascar, India, and Europe (Benton, 1984; Butler et al., 2015; Chatterjee, 1974; Dilkes, 1998; Ezcurra et al., 2020; Fitch et al., 2023; Langer et al., 2000, 2010; Montefeltro et al., 2013; Mukherjee & Ray, 2014; Nesbitt & Whatley, 2004; Schultz et al., 2016; Sues et al., 2020). Rhynchosaurs could reach up to two meters in length, with a body plan described as “pig‐like” (Ezcurra et al., 2020). Their skulls have beak‐like structures at the front of both upper and lower jaws, accompanied by a complex grinding dentition formed by multiple rows of marginal teeth (Butler et al., 2015; Ezcurra et al., 2020). The back of the skull is very expanded, especially in Late Triassic forms, which indicates the presence of enlarged jaw muscles and a strong bite (Ezcurra et al., 2020).

The multiple tooth rows of rhynchosaurs are a unique feature among stem‐archosaurs and are relatively rare in amniotes (e.g., captorhinids — de Ricqlès & Bolt, 1983; LeBlanc & Reisz, 2015). This provides a unique perspective on dental tissue formation and maintenance in a group of archosauromorphs that did not continuously replace their teeth but instead added new teeth at the back of the jaw, allowing individual teeth to remain in place for an extended period (Benton, 1984; Cabreira, 2004; Chatterjee, 1974; Sethapanichsakul et al., 2023). Furthermore, the rhynchosaur dentition has been described as “ankylothecodont” (see Mestriner et al. 2025 for a critical discussion of this terminology), which means that their teeth were implanted in deep sockets within the jaw, but contrary to conventional thecodonts (such as crocodylians, dinosaurs, and mammals), they were supposedly not held in place by a complex ligament‐based tooth attachment system (LeBlanc et al., 2017; LeBlanc et al., 2018). Instead, they were fused, or ankylosed, directly to the bone, as in many early amniotes (LeBlanc & Reisz, 2013; MacDougall et al., 2014; Maxwell, Caldwell, Lamoureux, & Budney, 2011), some early synapsids (LeBlanc et al., 2018), and some early dinosauriforms (Mestriner et al., 2022). However, recent investigations of the tooth attachment of many extinct amniotes have demonstrated the presence of mammal‐like periodontal tissues (alveolar bone, cellular cementum, and periodontal ligament; LeBlanc et al., 2018; Mestriner et al., 2022) even when teeth are ankylosed to the jaws. These studies have posited that differences between ankylosis and the mammal gomphosis are not due to differences in tooth attachment tissues types, but due to heterochronic shifts in the timing of mineralization of homologous periodontal tissues (LeBlanc et al., 2018; Mestriner et al., 2022). This means that, even when teeth are fully fused to the jaws, histological analyses should reveal the presence of cementum, alveolar bone, and a mineralized periodontal ligament, rather than a single “bone of attachment” tissue.

The tissues responsible for ankylosing rhynchosaur teeth are still debated (Sethapanichsakul et al., 2023). In the literature, their ankylosis has been associated with a so‐called “bone of attachment” (Benton, 1984; Cabreira, 2004; Chatterjee, 1974), an organization that would differ from the tissues that we now know are responsible for fusing the teeth to the jaws in other “ankylothecodont” archosaurs, such as silesaurids (Mestriner et al., 2022). The specimens analyzed here are referred to *Stenaulorhynchus stockleyi*, the only rhynchosaur currently recorded in the Middle Triassic Manda Beds of Tanzania. This species is characterized by a maxilla with three tooth rows and lingual teeth, and a dentary bearing two longitudinal rows and lingual teeth. Furthermore, we conducted a detailed histological analysis of this species in order to assess rhynchosaur ankylothecodonty in the broader evolutionary context of dental tissue evolution across Archosauromorpha.

## 2 METHODS

Three specimens of *Stenaulorhynchus stockleyi* (rhynchosaur) were thin‐sectioned at the ROM (Royal Ontario Museum, Toronto, Canada) Palaeohistology Laboratory. All paleohistological sections followed the standard procedures employed for sectioning fossil material according to LeBlanc et al. (2018). In order to retain the original shape of the specimens, CT scans, molds, and casts were obtained before thin sectioning. CT scan resolution and contrast were not sufficient to resolve histological data and were, therefore, not incorporated into this study. The specimens were individually embedded in polyester resin within a plastic container. Resin hardener was added at a ratio of 9 mL per 18 mL of polyester resin. The samples were then placed under vacuum for approximately 5 min to remove air bubbles and allowed to cure for 24 h. Once set, the specimens were sectioned using a Buehler Isomet 1000 water blade saw operating at a speed of 200–300 rpm. The cut surfaces were ground and polished with 600‐ and 1,000‐grit silicon carbide powder in suspension on a glass plate. They were subsequently mounted onto frosted plexiglass slides using Scotch‐Weld SF‐100 cyanoacrylate glue. After mounting the specimens to the slides, thick sections approximately 0.7 mm in thickness were trimmed using the Isomet 1000 saw. These sections were further thinned and smoothed using a Hillquist grinding machine, with regular microscopic inspection to monitor progress. Final grinding was done manually on a glass plate with 600‐ and 1,000‐grit silicon carbide powder until the sections reached near‐optical clarity. The specimens were polished to completion using 1‐μm aluminum oxide powder and a soft cloth. Thin section images were captured using a fully automated Nikon AZ100 microscope system and an E200 polarizing petrographic microscope. Image acquisition was performed using Nikon NIS Elements imaging software. The original slides and remaining embedded specimens are temporarily available at Virginia Tech University (NMT RB1627 and NMT RB1628) and the Burke Museum (NMTRB 95), but will be permanently reposited at the National Museum of Tanzania in Dar es Salaam (Sidor & Nesbitt, 2018).

## 3 MATERIAL

**FIGURE 1:**
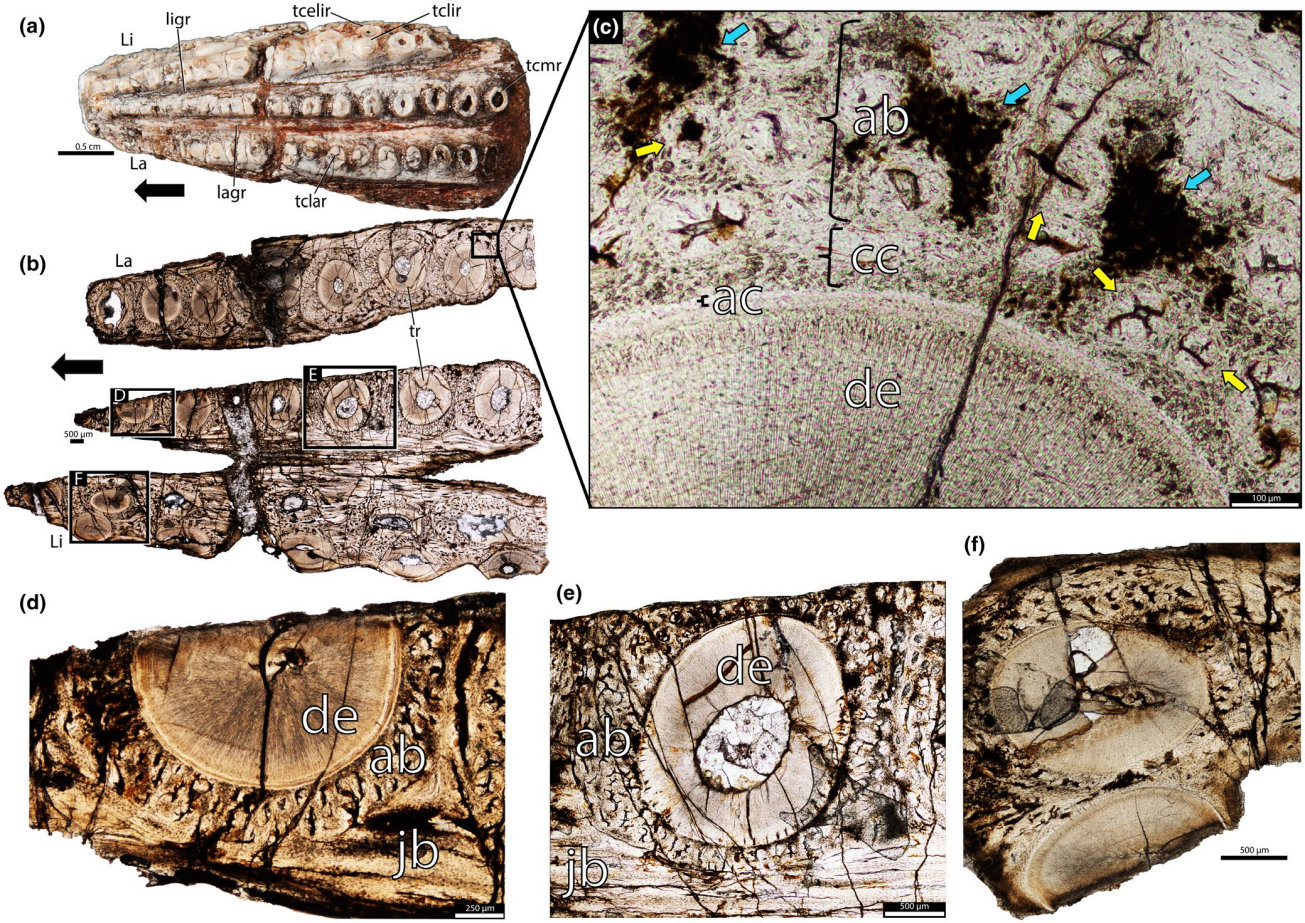
Right maxilla of *Stenaulorhynchus stockleyi* (NMT RB95). (a) Ventral view of the specimen before thin sectioning, showing tooth rows position. (b) Transversal thin section of the tooth rows, detailing tooth position. (c) Close‐up view with details of tooth attachment, showing the alveolar bone contacting the cellular cementum. (d, e) Details of the connection between alveolar bone and jawbone. (f) Details of teeth from two different rows contacting each other. ab, alveolar bone; ac, acellular cementum; cc, cellular cementum; de, dentine; jb, jawbone; lagap, labial gap; ligap, lingual gap; La, labial; Li, lingual; lagr, labial groove; ligr, lingual groove; tcelir, tooth crown extra lingual row; tclar, tooth crown labial row; tclir, tooth crown lingual row; tcmr, tooth crown middle row; tr, tooth roots. Black arrows indicate anterior direction. Blue arrows, dark areas show accumulations of osteocytes; Yellow arrows, lamellar bone along the vascular channel walls.

**FIGURE 2:**
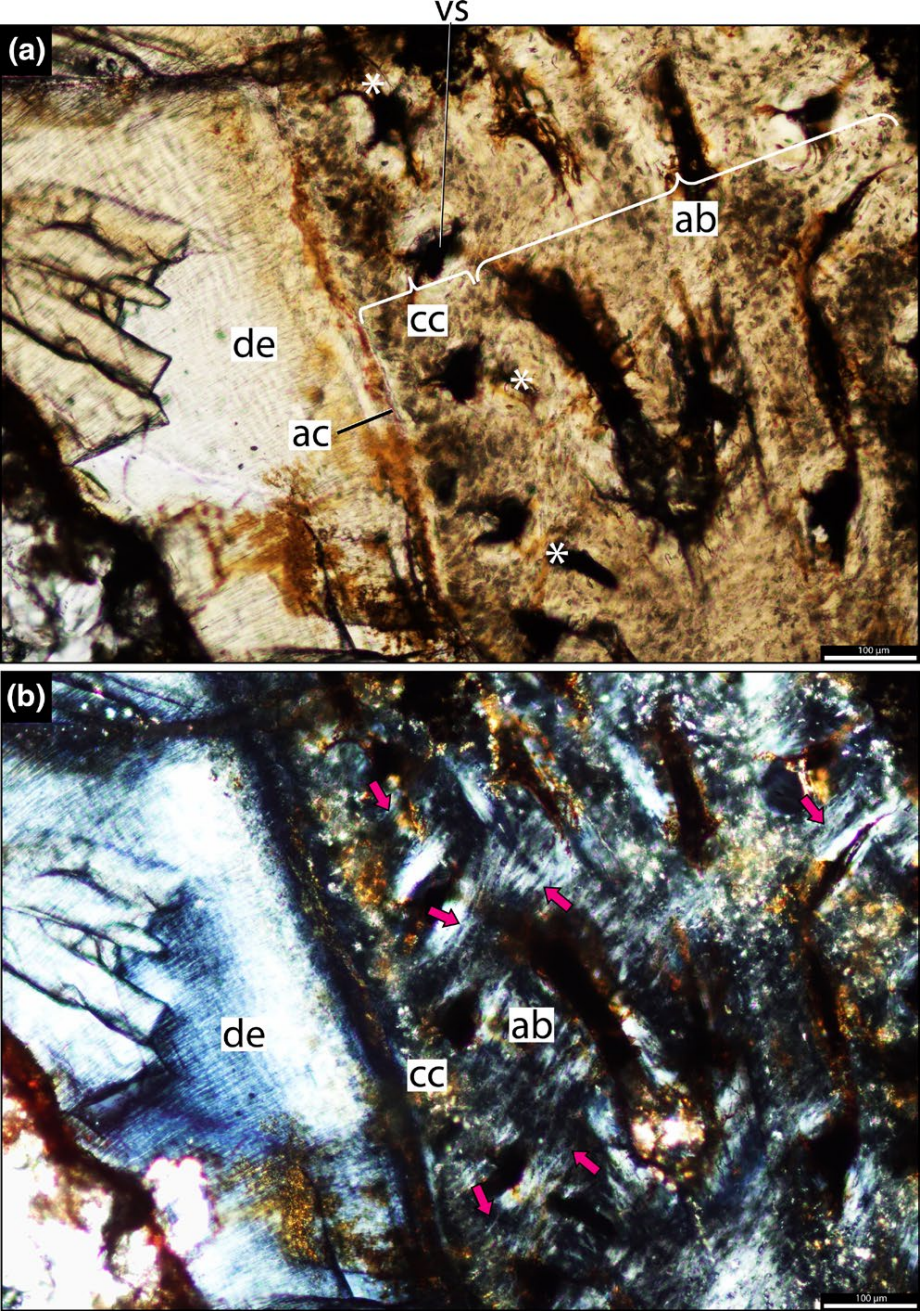
Histology of the right maxilla of *Stenaulorhynchus stockleyi* (NMT RB95). Dental tissues in coronal section at root level. Dorsal is toward the top of the page. (a) Tooth attachment details showing contact between alveolar bone and cellular cementum (mineralized periodontal space/ankylosis). (b) Panel (a) viewed under cross‐polarized light, showing Sharpey’s fibers across the alveolar bone and cementum layers. Asterisks highlight where the cellular cementum and alveolar bone are in contact. ab, alveolar bone; ac, acellular cementum; cc, cellular cementum; de, dentine; vs, vessel space. Pink arrows, Sharpey’s fibers.

**FIGURE 3:**
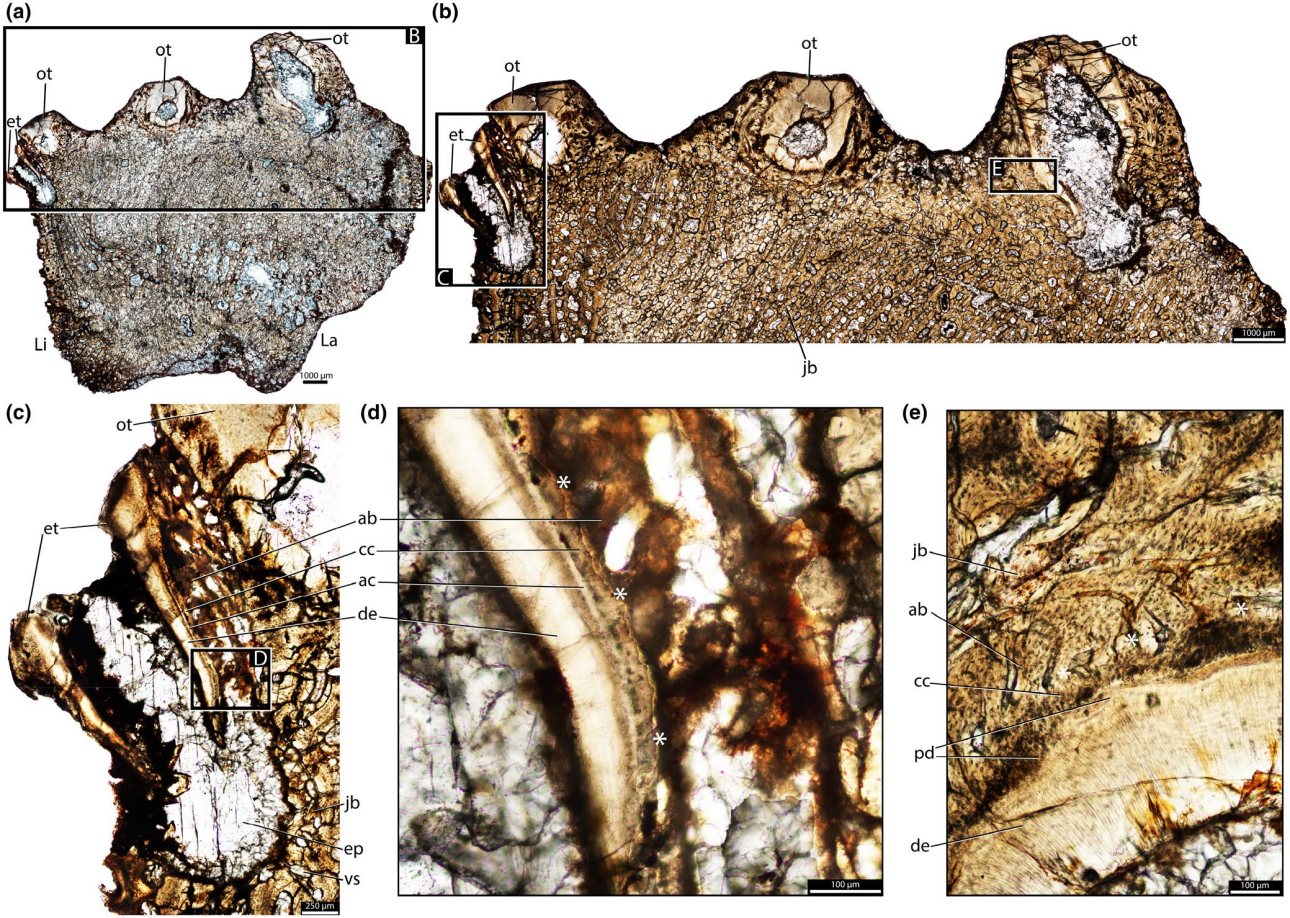
Histology of the right maxilla of *Stenaulorhynchus stockleyi* (NMT RB95). Dental tissues in coronal section. (a) General view of three tooth rows and maxillary jawbone. (b) Higher magnification view of the tooth roots in each row. (c, d) Tooth attachment details of an emplacement tooth in the lingual row — in the process of being emplaced, showing the alveolar bone contacting the cellular cementum. (e) Tooth attachment details of the tooth positioned in the labial row, rotated 90 degrees clockwise for comparisons with the others. Asterisks highlight contacts between cellular cementum and alveolar bone. ab, alveolar bone; ac, acellular cementum; cc, cellular cementum; de, dentine; ep, emplacement pit; et, emplacement tooth; jb, jawbone; La, labial; Li, lingual; ot, old tooth; pd, plicidentine; vs, vessel space.

NMTRB95 (Figure S1) is a right maxilla missing the rostral and caudal portions, but the tooth tissues are well‐preserved (Figures 1, 2, 3). The teeth are conical, with the enamel caps missing. There are three main longitudinal tooth rows, separated by grooves and forming an elongated occlusal surface. An extra row is present on the lingual side, close to the most lingual of the main rows, whereas additional teeth are scattered along the lingual surface. Tooth size increases caudally, and the number of teeth varies between rows (Figure 1a). From labial to lingual, the rows include 13, 12, 10, and 8 teeth. Some areas exhibit extensive tooth wear, with teeth appearing completely worn down rather than broken away post‐mortem.

**FIGURE 4:**
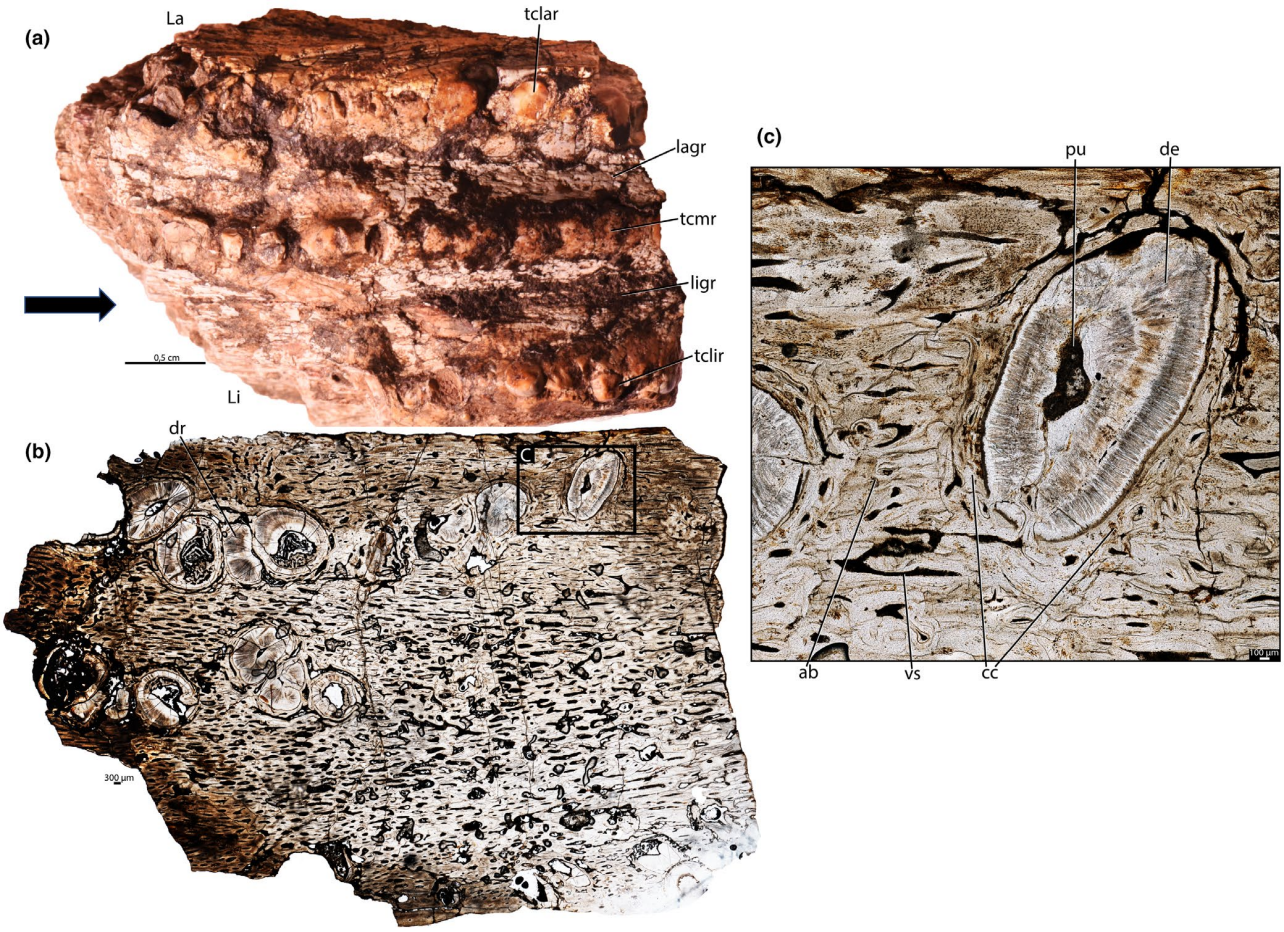
Right maxilla of *Stenaulorhynchus stockleyi* (NMT RB1627). (a) Ventral view of the specimen before thin sectioning, showing tooth rows positions. (b) General transverse view of the dental tissues at root level. (c) Approximate view showing tooth attachment details. ab, alveolar bone; cc, cellular cementum; de, dentine; dr, dentine remnant; La, labial; Li, lingual; lagr, labial groove; ligr, lingual groove; pu, pulp cavity; tclar, tooth crown labial row; tclir, tooth crown lingual row; tcmr, tooth crown middle row; vs, vessel space. Black arrow indicates anterior direction.

NMT RB1627 (Figure S2) is a right maxilla missing its rostral portion, with several cracked and crushed teeth preserved (Figure 4). The teeth are conical, with enamel caps mostly lost. There are three main tooth rows, separated by longitudinal grooves, forming an elongated occlusal surface. An additional row of teeth is present lingually, misaligned with the main rows (Figure 4a), and tooth size decreases rostrally (Figure 4a). The lingual row contains seven crowns, whereas the middle row has nine, and the labial row has approximately thirteen. All rows display evidence of wear, with most preserved tooth crowns being abraded.

**FIGURE 5:**
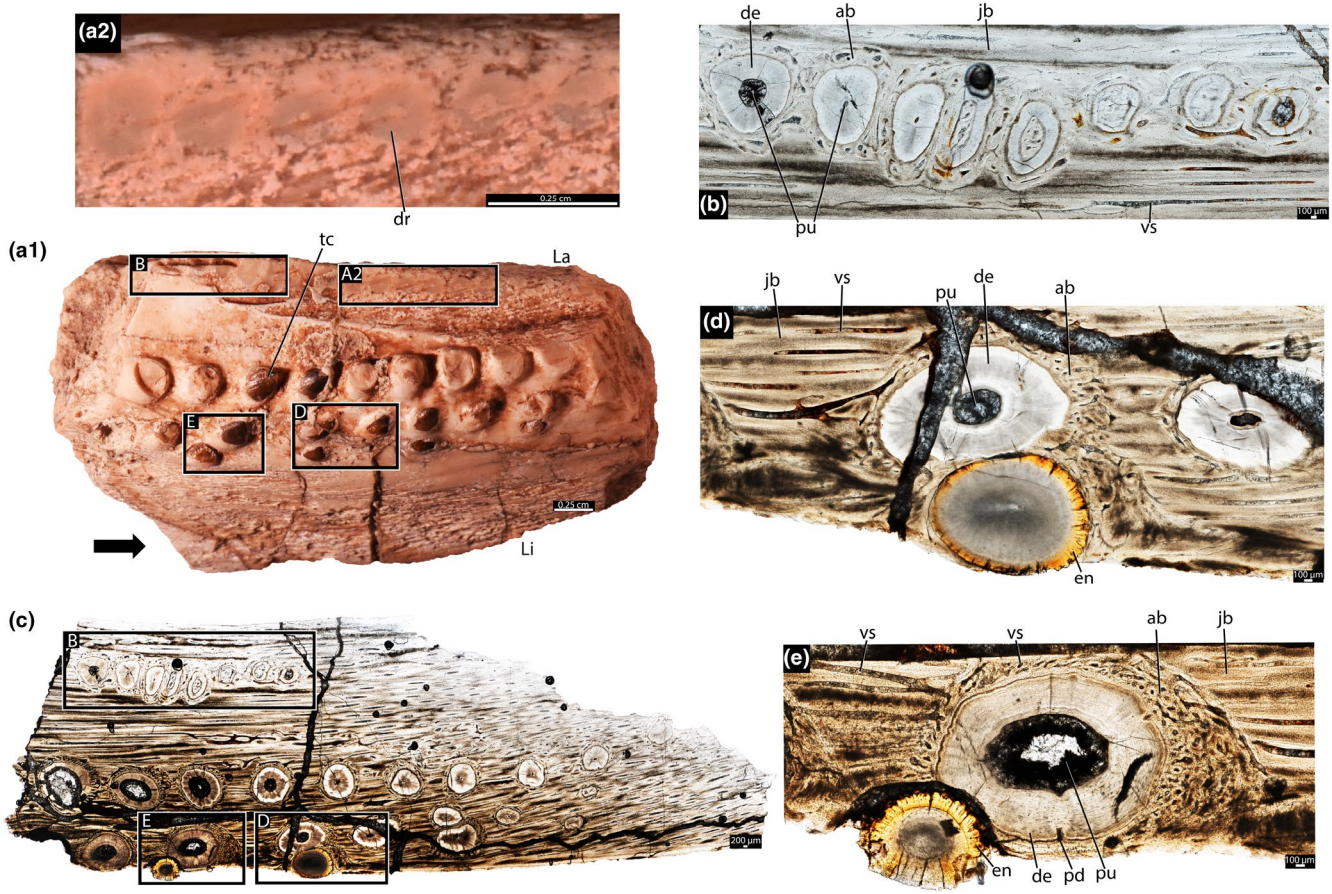
Left dentary of *Stenaulorhynchus stockleyi* (NMT RB1628). (a1) Dorsal view of the specimen before thin sectioning, showing tooth row positions. (a2) Close‐up of the labial tooth row showing impressions of teeth (remnants of dentine) that have worn out their crown. (b) Transverse (horizontal) section showing a row of teeth at root level that also had their crowns worn out at the surface, similar to panel (a2). (c) General view of the dental tissues in transverse (horizontal) section at root level. (d, e) Higher magnification views showing the contact between some of the teeth shown in panel (c) and additional details of tooth attachment. ab, alveolar bone; de, dentine; dr, dentine remnant; en, enamel; jb, jawbone; La, labial; Li, lingual; pd, plicidentine; pu, pulp cavity; tc, tooth crown; vs, vessel space. Note that “c”, “d” and “e” are reversed in order to align to “a”. Black arrow indicates the anterior direction.

**FIGURE 6:**
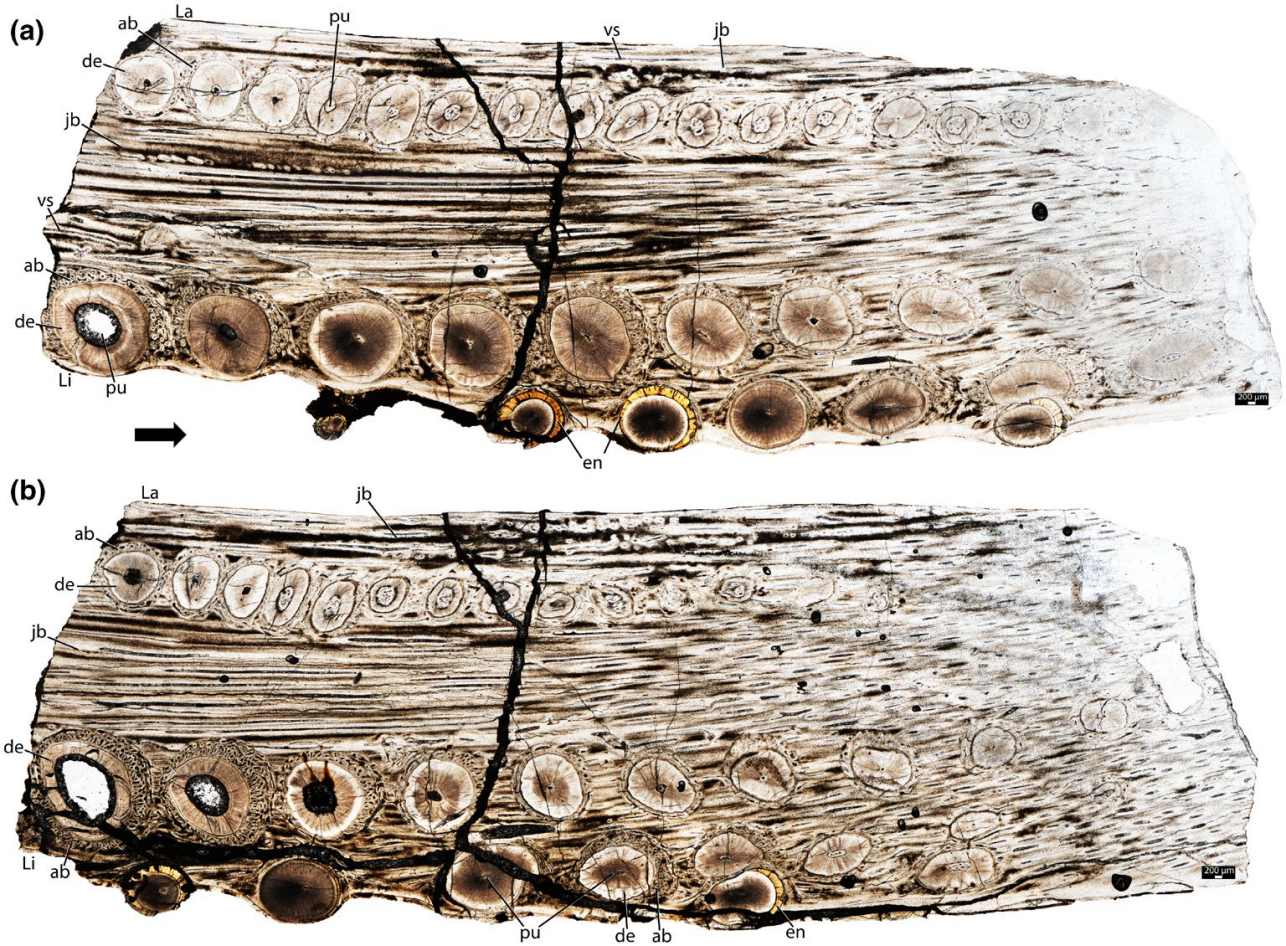
Histology of the left dentary of *Stenaulorhynchus stockleyi* (NMT RB1628). General view of dental tissues in two different transverse sections at the level of the tooth roots, showing three tooth rows. (a) Section closer to the tooth crown. (b) Section closer to the apex of the tooth root. Note that “Figure 5c” corresponds to a section that is more ventral than the one shown in “Figure 6b”. ab, alveolar bone; cc, cellular cementum; de, dentine; en, enamel; jb, jawbone; La, labial; Li, lingual; pu, pulp cavity; vs, vessel space. Black arrow indicates the anterior direction.

**FIGURE 7:**
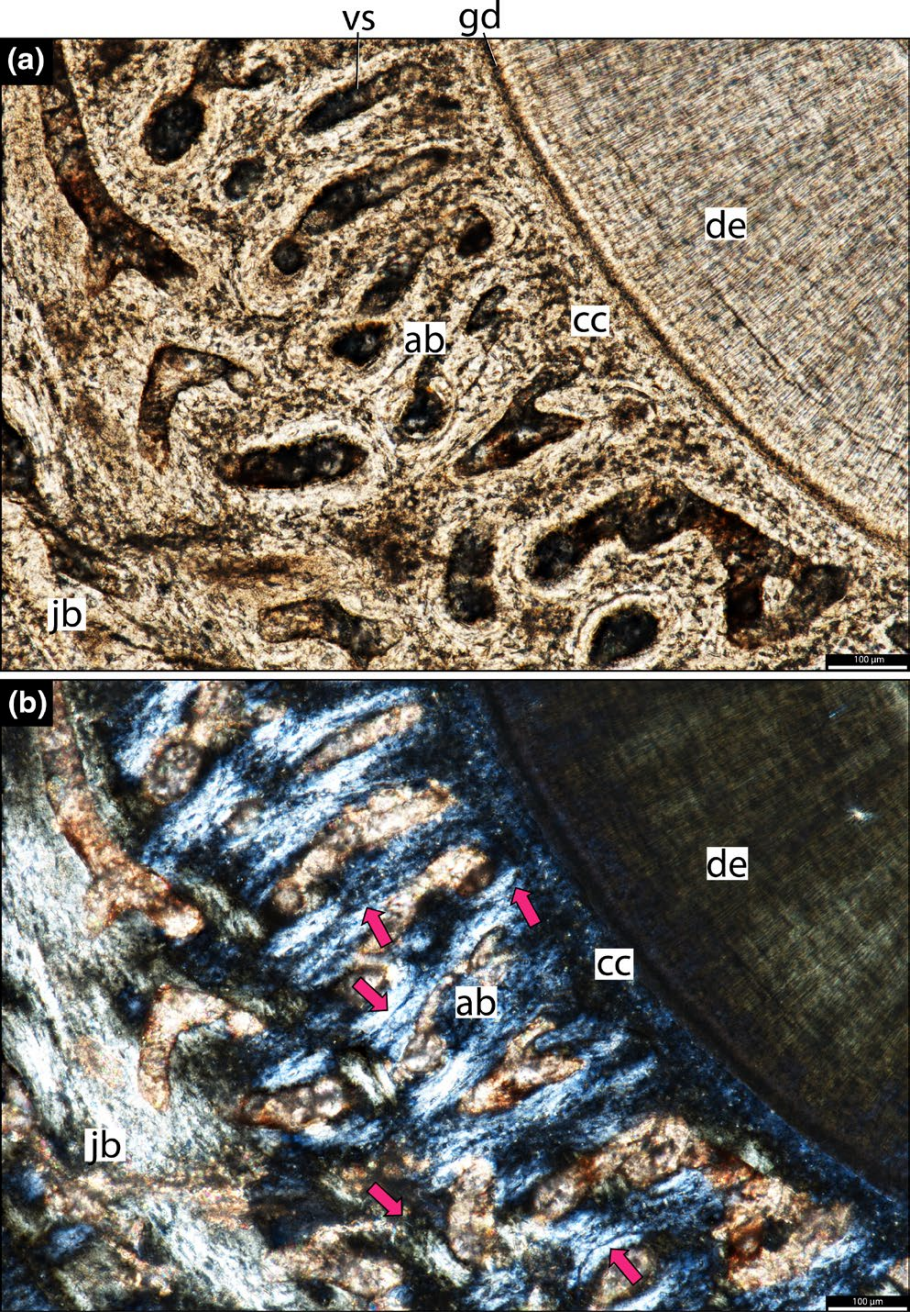
Histology of the left dentary of *Stenaulorhynchus stockleyi* (NMT RB1628). Dental tissues in transverse section at root level. (a) Tooth attachment details showing contact between alveolar bone and cellular cementum (mineralized periodontal space/ankylosis). (b) Panel (a) viewed under cross‐polarized light, showing Sharpey’s fibers across the alveolar bone and cementum layers. ab, alveolar bone; cc, cellular cementum; de, dentine; gd, granular dentine; jb, jawbone; vs, vessel space. Pink arrows, Sharpey's fibers.

**FIGURE 8:**
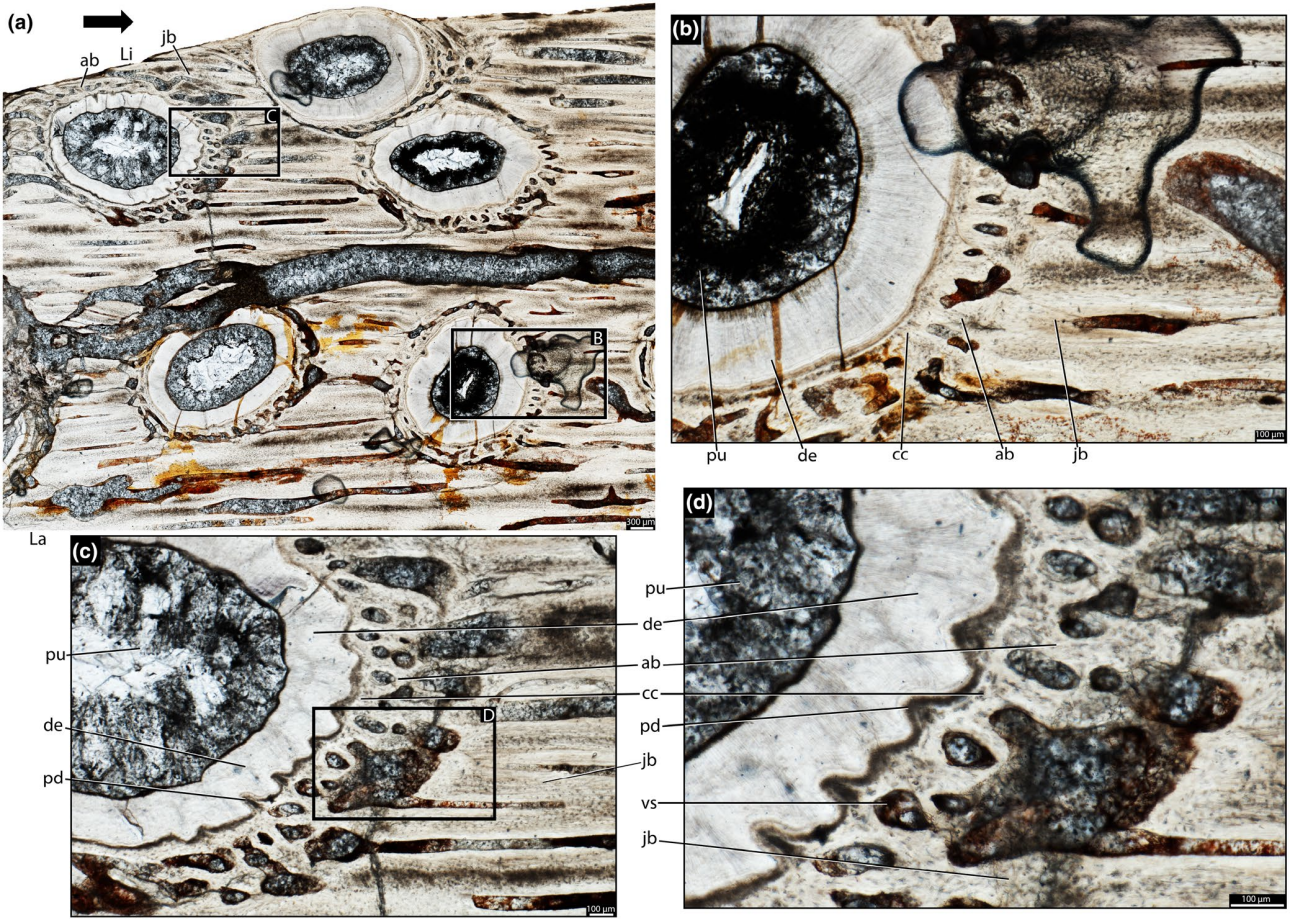
Histology of the left dentary of *Stenaulorhynchus stockleyi* (NMT RB1628). Dental tissues in transverse section at root level. (a) Tooth rows showing jawbone between teeth. (b) Tooth attachment detail (labial tooth row), showing contact between alveolar bone and cellular cementum (mineralized periodontal space/ankylosis), and between jawbone and alveolar bone. (c) Tooth attachment detail (lingual tooth row), showing contact between alveolar bone and cellular cementum (ankylosis), and between jawbone and alveolar bone. (d) Close‐up of the attachment tissues shown in panel (c), highlighting the presence of plicidentine. ab, alveolar bone; cc, cellular cementum; de, dentine; La, labial; Li, Lingual; jb, jawbone; pd, plicidentine; pu, pulp cavity; vs, vessel space. Black arrow indicates the anterior position.

**FIGURE 9:**
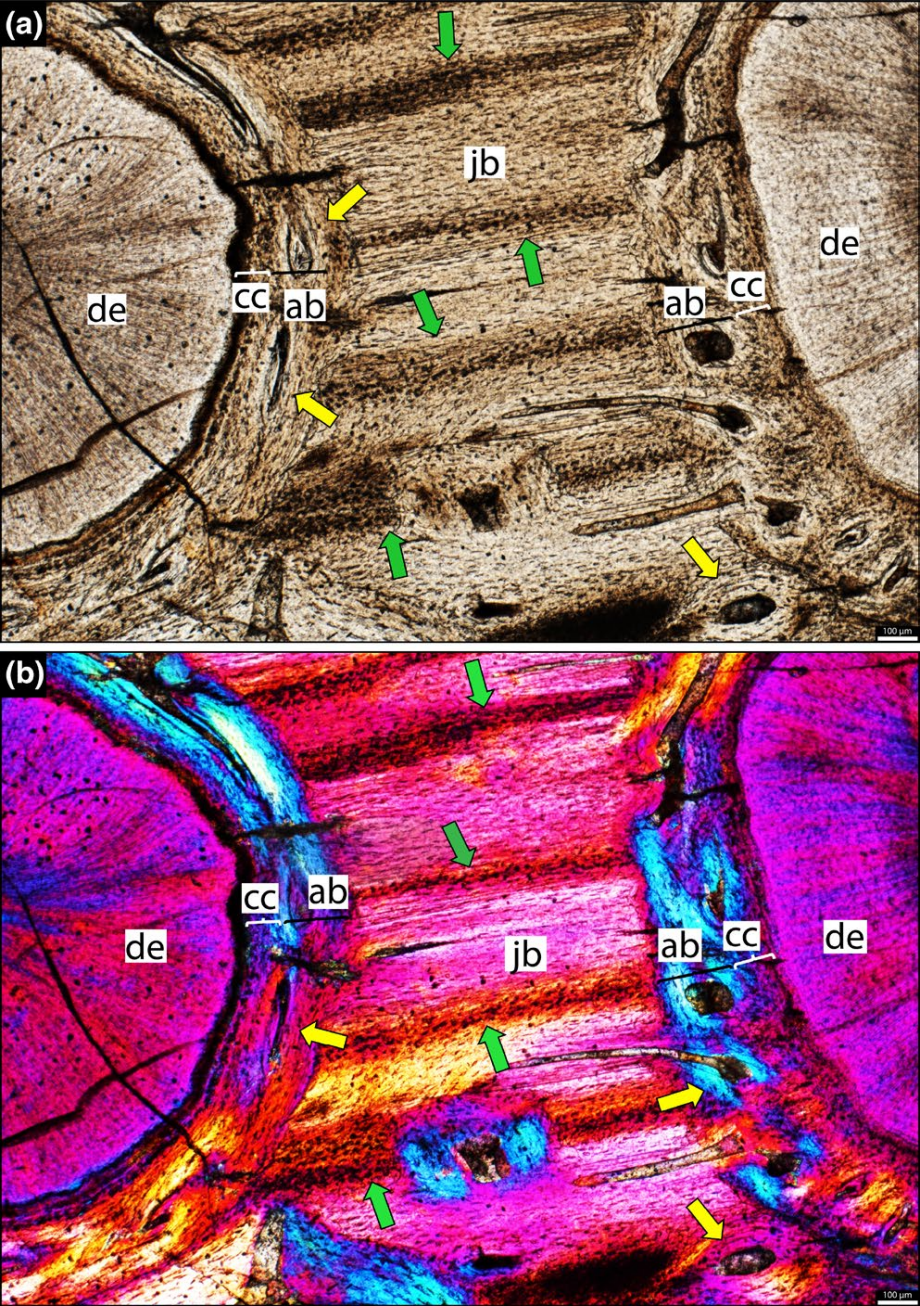
Histology of the left dentary of *Stenaulorhynchus stockleyi* (NMT RB1628). Dental tissues in transverse section at the root level. (a) Tooth attachment details showing contact between alveolar bone and cellular cementum (mineralized periodontal space/ankylosis), and a row of accumulated osteocytes in the jawbone, contacting both the alveolar bone and cellular cementum. (b) Panel (a) viewed under lambda‐filtered cross‐polarized light, showing the same structures with different contrast. ab, alveolar bone; cc, cellular cementum; de, dentine; jb, jawbone. Green arrows, osteocytes accumulation. Yellow arrows, lamellar bone along the vascular channel walls.

**FIGURE 10:**
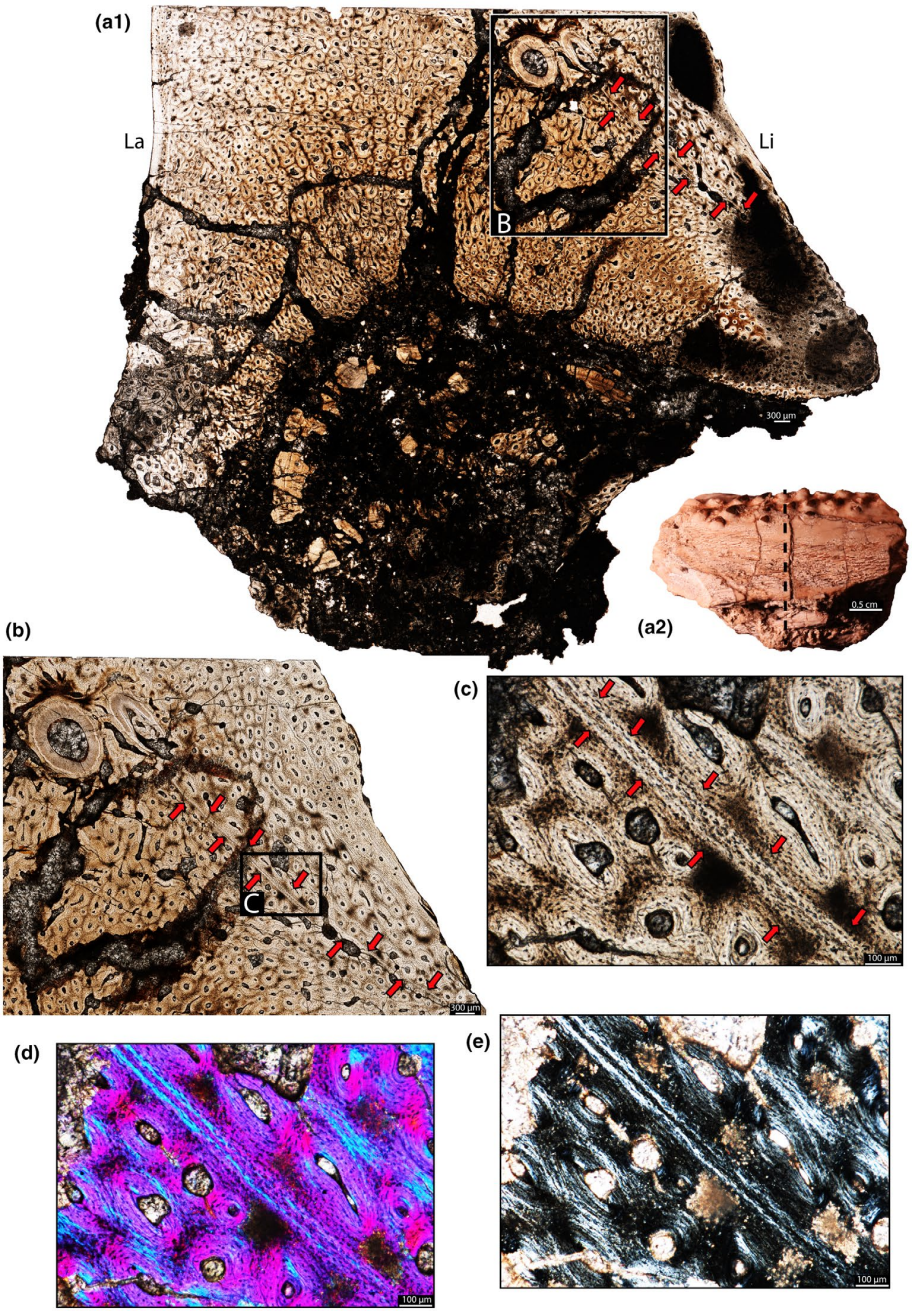
Histology of the left dentary of *Stenaulorhynchus stockleyi* (NMT RB1628). Dental tissues in coronal section. Dorsal is toward the top of the page. (a1) General view of the jaw, showing two relict, non‐functional partial teeth (Panel b) buried deeper within the jawbone. (a2) Specimen in lingual view prior to thin sectioning, showing the approximate location of the cut (dashed line). Anterior direction is toward the right side of the page. (b) Close‐up of the two relict teeth, and an extensive band of osteocyte. (c) Detail of the osteocyte band. (d, e) Same as panel (c), viewed under lambda‐filtered cross‐polarized light and standard cross‐polarized light, respectively. Red arrows, osteocyte band.

**FIGURE 11:**
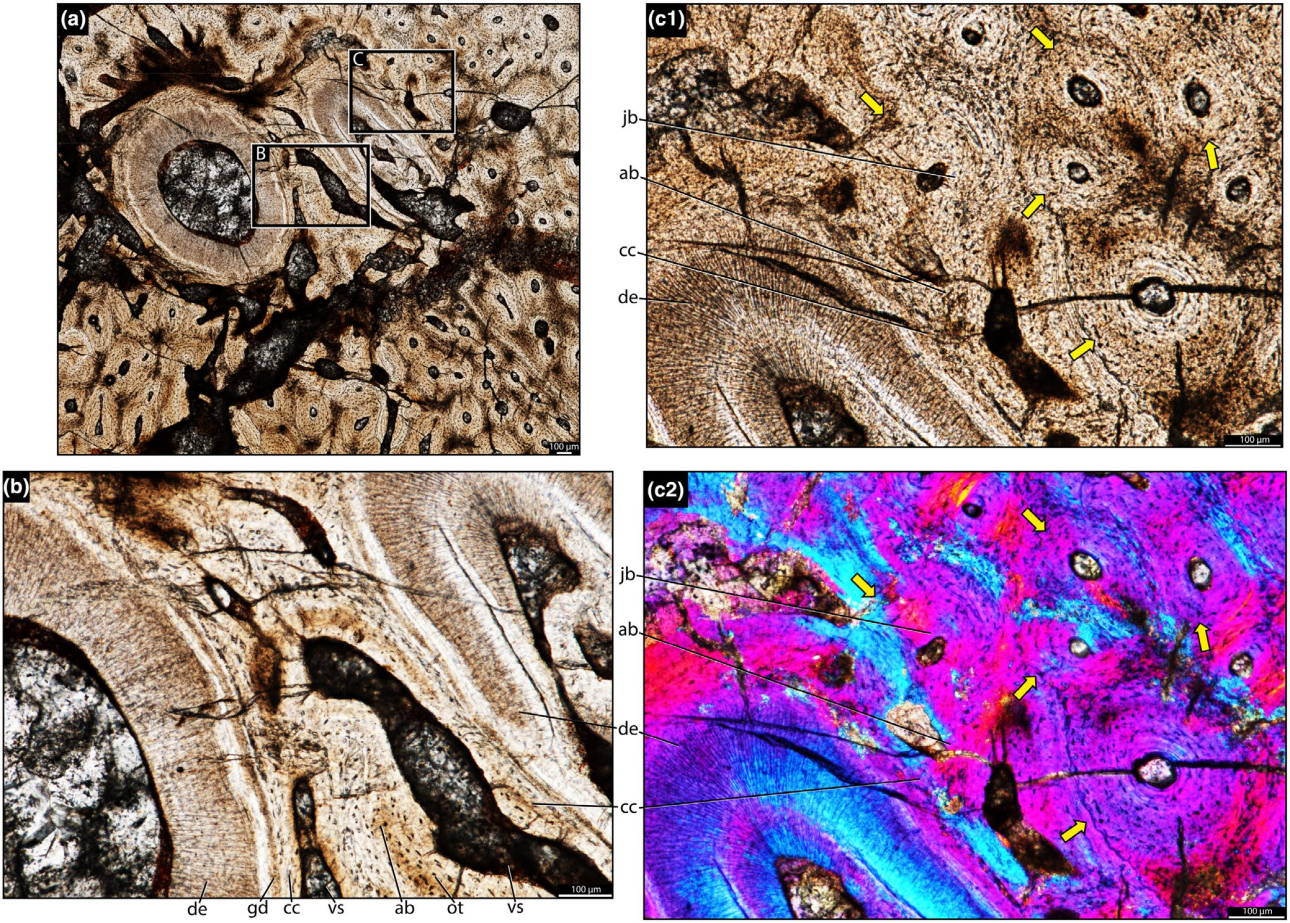
Histology of the left dentary of *Stenaulorhynchus stockleyi* (NMT RB1628). (a) Tooth attachment details of the two relict teeth from “Figure 10”, in coronal section. (b) Close‐up of an area in panel (a) showing the contact between alveolar bone and cellular cementum. (c1) Close‐up of another area in panel (a), showing alveolar bone and cellular cementum in contact, and the presence of lamellar bone along the vascular channels. (c2) Same as panel (c1), viewed under lambda‐filtered cross‐polarized light. ab, alveolar bone; cc, cellular cementum; de, dentine; gd, granular dentine; jb, jawbone; ot, osteocyte; vs, vascular space. Yellow arrows, lamellar bone along the vascular channel walls.

NMT RB1628 (Figure S3) is a left dentary missing its rostral and caudal portions, but the teeth are well‐preserved (Figures 5, 6, 7, 8, 9, 10, 11). Two main tooth rows dominate the lingual side of the jaw, and a third row, with fewer teeth, is positioned more ventrolingually (Figures 5 and 6). Labially, a fourth longitudinal row of smaller teeth is present (Figures 5a,b and 6). The crowns of this labial row are entirely worn‐out, leaving only their cross sections — most of which are ovoid, with the long axes oriented labiolingually — on the jaw surface, where the roots are still preserved. In the more lingual rows, the rostral teeth are conical and lack enamel, whereas the caudal ones are triangular (“pencil‐shaped” sensu Sethapanichsakul et al., 2023) and less worn, retaining their enamel caps. In the first of the two main lingual rows, ten tooth roots are visible in thin sections, but only nine crowns are exposed. The second main row bears eight roots (Figure 6b), but only seven crowns are exposed, whereas the extra row has only four teeth (Figure 5a1). Some tooth roots show a golden outline around the dentine, which corresponds to enamel (Figures 5d,e and 6). Tooth size increases progressively from the rostral to the caudal regions in all rows (Figures 5 and 6).

## 4 RESULTS

Rhynchosaur teeth are deeply implanted within the jaws (Figure 3) and possess low, usually heavily worn crowns (Figures 1a, 4a and 5a). The caudal portions of the dentary and maxilla are labiolingually wider and exhibit more tooth rows (Figures 1a, 4a and 5a). We found evidence of partial tooth resorption between several neighboring teeth (Figures 4b, 5d, and e); however, we did not observe the one‐for‐one tooth replacement mode that is typical of polyphyodont amniotes (Edmund, 1960; Fong et al., 2016; LeBlanc et al., 2017). New teeth partially resorbed the attachment tissues and dentine of adjacent teeth, but this did not lead to shedding of older teeth. Instead, new teeth seem to have been constantly added to tooth rows, similar to Palaeozoic captorhinid reptiles (de Ricqlès & Bolt, 1983; LeBlanc & Reisz, 2015) and some multiple tooth‐rowed therapsids (Olroyd et al., 2021). In the absence of tooth shedding, the crowns were worn down flush with the surrounding bone (leaving only a flat trace on the surface; Figure 5a2), and the roots of the teeth remained intact (Figure 5b).

The dentine is composed of long tubules radiating from the pulp cavity towards the external surface. Successive incremental growth lines perpendicular to the tubules are visible in some of the best preserved teeth (Figures 5e and 7a). These lines have a wave‐like pattern that mirrors the external dentine contour in transverse (horizontal) sections (Figure 5e). In some teeth, the dentine has localized infoldings that we interpret as shallow dentine folds, i.e., plicidentine (Figures 3e, 5e, 8 and 9; Kearney & Rieppel, 2006; Maxwell, Caldwell, Lamoureux, & Budney, 2011; Maxwell, Caldwell, & Lamoureux, 2011c; Brink et al., 2014; MacDougall et al., 2014; Palci et al., 2021). Unlike the deep and radial plicidentine infoldings seen in fishes (Viviani et al., 2022), early amniotes (Maxwell, Caldwell, & Lamoureux, 2011c), varanids and venomous snakes (Palci et al., 2021), plicidentine in rhynchosaurs forms shallow infoldings along the periphery of the tooth roots (Figures 3e, 5e and 8d). Additionally, we observed that these dentine infoldings are consistently found at different levels in the transverse sections, indicating that they extended from the tooth base almost to the crown, but were very subtle near the crown (Figure 5d,e). The pulp cavity itself varies in shape and size, ranging from small, nearly enclosed ovoid or circular cavities (Figures 4c, 5e, and 6) to larger, rounded cavities (Figure 6). Globular dentine separates the orthodentine from the root cementum (Figures 7 and 11b), forming the granular layer of Tomes (Nanci, 2013), a feature also observed in neotheropod dinosaurs (Fong et al., 2016) and silesaurids (Mestriner et al., 2022). Externally, a thin (~13.40 μm) layer of acellular cementum lies adjacent to the globular dentine. This layer is distinct from the overlying cellular cementum due to its lighter color (Figures 1c, 2a, 3c,d). Cellular cementum, which is thicker (~96 μm), bears abundant cementocyte spaces within a disorganized matrix that is distinct from the surrounding bone under cross‐polarized light (Figure 7b), resembling that found in other archosaurs (Fong et al., 2016; LeBlanc et al., 2017; Mestriner et al., 2022). Cellular cementum in rhynchosaurs can be vascularized (Figure 2) or avascular (Figures 3, 8 and 9). Sharpey’s fibers perforate the cellular cementum and extend circumferentially into the alveolar bone (Figures 2b and 7b). The interface between cellular cementum and alveolar bone often appears darker under white light compared with surrounding tissues, owing to a higher density of Sharpey's fibers (Figure 1c,d).

The alveoli are composed of a thick (~850 μm in some regions) layer of a highly vascularized woven fiber alveolar bone (Figures 1, 2, 3, 4, 5, 6, 7, 8, 9, 10, 11), with simple oval vascular channels (Figures 7, 8), typical of early vertebrates (Caldwell et al., 2003; Peyer, 1968), including archosaurs (Fong et al., 2016; LeBlanc et al., 2017; Mestriner et al., 2022). However, as rhynchosaur teeth mature, and given the absence of replacement events, lamellar bone starts to be added along the vascular channel walls, forming primary osteons around the root (yellow arrows in Figures 1c, 9, and 11c), which resemble the lamellar structure forming mammal tooth sockets (LeBlanc et al., 2018). In rhynchosaurs, these vascular channels lie external to the contact with the cellular cementum, as in early dinosauriforms (Mestriner et al., 2022). The teeth are ankylosed, with full contact between the alveolar bone and the cellular cementum (Figures 1, 2, 3 and 7, 8, 9). The region between adjacent teeth predominantly consists of jawbone (Figures 8 and 9). This contrasts with the alveolar bone‐dominated “interdental bone” of crocodylians and dinosauriforms, including dinosaurs (Fong et al., 2016; LeBlanc, 2023; LeBlanc et al., 2018; Mestriner et al., 2022). However, it is worth mentioning that in NMT 1628, the middle row of teeth in the caudal region has a larger area of alveolar bone with a clearly defined circular boundary, where only a small amount of jawbone is wedged in between tooth positions (Figure 6). The jawbone in rhynchosaurs is parallel‐fibered and more extensive than alveolar bone (Figure 6). The vascular channels in the jawbone differ from those of alveolar bone in being mesiodistally elongated and labiolingually narrow in transverse views (Figures 5d,e, 6 and 8). In coronal view, the vascular spaces appear rounded and organized in dorsoventral bands (Figures 3 and 10, 11). The region between adjacent teeth exhibits dark bands of accumulated osteocytes, visible in both transverse (Figure 6, and green arrows in Figure 9) and coronal views (Figure 3). In coronal sections, these bands are dorsoventrally aligned and situated between the vascular bands (e.g., Figure 3c). Transversally, dark bands are also present in regions where the jawbone contacts the alveolar bone, marked by a reversal line outlining each tooth (Figures 1d–f and 9). This line delineates the boundary between the jawbone and the alveolar bone (LeBlanc et al., 2017), with evidence of remodeling activity at the distal ends (closer to the jawbone) of the alveolar bone. Additionally, osteocyte‐dense dark spots are observed in the alveolar bone of some teeth (blue arrows in Figure 1c).

In a coronal section of NMT RB1628, we observed two relict, non‐functional partial teeth buried in the dentary at a deeper level (Figures 10 and 11), which probably occurred as a result of bone growth. Ventral to these teeth, there is a thick perpendicular tissue band (~100 μm) with parallel‐fibered bone matrix and osteocyte lacunae dorsoventrally aligned into layers (red arrows in Figure 10). This band extends dorsally from the ventral portion of the dentary (ventrally) to near the base of those teeth at the top. This structure appears to represent a growth line, indicating the direction in which bone tissue of the dentary was added. Additionally, the more labial tooth exhibits a peculiar morphology, where instead of the expected conical shape for a tooth in this orientation (e.g., Figure 3), it has an ovoid shape, resembling the morphology of teeth in dorsoventral view.

## 5 DISCUSSION

### 5.1 Ankylothecodonty in rhynchosaurs

The “ankylothecodonty” of rhynchosaurs was first interpreted as teeth being firmly ankylosed by a spongy‐like “bone of attachment” surrounding the socketed portion of each tooth (Chatterjee, 1974). Subsequent studies also failed to identify mammal‐like tooth attachment tissues in the group and, as a consequence, “bone of attachment” became the standard term to describe the tissue responsible for fusing the teeth to the jaws (Benton, 1984; Cabreira, 2004). This echoes the historical assumption that across non‐mammalian, non‐crocodylian amniotes, teeth are typically fused in place via a homogeneous “bone of attachment” tissue (Peyer, 1968; Tomes, 1882).

Recent analyses using CT scan data challenged the presence of “bone of attachment” in rhynchosaur (Sethapanichsakul et al., 2023), but found no evidence of ankylosis mediated by alveolar bone, persistent sockets, or gomphosis via a periodontal ligament. Although no trace was found, that study did not rule out the possible presence of alveolar bone during the initial stages of implantation, particularly where presumable resorption pits occurred on the bone surface (Sethapanichsakul et al., 2023). Our histological data reveal that rhynchosaur teeth do indeed bear mammal‐like attachment tissues—acellular and cellular cementum, and alveolar bone (Figures 2, 4, and 7, 8, 9)—which are now generally considered plesiomorphic for Amniota (LeBlanc & Reisz, 2013; Fong et al., 2016; LeBlanc et al., 2017, LeBlanc, 2023; Mestriner et al., 2022, 2025). Moreover, we interpret the presence of a periodontal ligament (PDL) based on the occurrence of radiating Sharpey’s fibers around fully ankylosed teeth (Figures 2b and 7b). These represent the insertion points of a formerly soft collagen fiber bundle system (the unmineralized PDL) that eventually becomes completely mineralized through growth of alveolar bone and cementum.

We also show that the rhynchosaur periodontium comprises persistent tooth sockets formed by highly vascularized alveolar bone with a woven fiber matrix (Figures 3, 7 and 8). However, unlike in crocodylians, dinosaurs, and mammals, their teeth were not placed within a dental groove but housed within discrete emplacement pits within the pre‐existing jawbone (Figure 3) (LeBlanc et al., 2017, 2018; Mestriner et al., 2022). For this to occur, osteoclasts would have to resorb a small portion of the jawbone in order to accommodate a developing tooth at the end of a row. These new emplacement teeth would be suspended within this pit by soft tissues, which would then quickly mineralize. Figure 3 shows this process occurring, with a tooth being emplaced in the jaw as the newest alveolar bone begins to develop, and just barely contacts the cellular cementum coating the tooth root (Figure 3c,d). Underneath the tooth, the resorbed area (emplacement pit — Figure 3c) represents the farthest extent of jawbone resorption to accommodate this new tooth. The connection between cellular cementum and alveolar bone is weak (Figure 3d) when compared to older and already emplaced teeth (e.g., Figures 7, 8, and 9), but the presence of ankylosis already in this initial stage indicates a relatively quick mineralization of the periodontal ligaments. Emplacement pits can also be observed externally in the lingual side of the rhynchosaur specimens (“ep” in Figures S1, S2), and their presence and positions confirm that, as for most amniotes, their teeth have an extraosseous origin and the odontogenetic organ (dental lamina) is positioned near the jaw surface (Edmund, 1960; Fong et al., 2016; Wu et al., 2013) rather than being disconnected from the surface and buried deep in the jaw, as in extant crocodylians (Fong et al., 2016; LeBlanc et al., 2017; Martin & Stewart, 1999). This condition is reminiscent of tooth emplacement and attachment in another reptile group with multiple tooth rows, the captorhinids (LeBlanc & Reisz, 2015).

The large amount of jawbone found between adjacent rhynchosaur teeth (Figures 3b, 6, 8, and 9) contrasts with the condition observed in archosaurs (Fong et al., 2016; LeBlanc et al., 2018; Mestriner et al., 2022), where it is mostly composed of alveolar bone, i.e., “interdental bone” (LeBlanc et al., 2023). The presence of abundant jawbone between successive generations of teeth in rhynchosaurs may be related to the prolonged interval between the formation of new teeth. This inference of a prolonged interval is predicated on the jaw first growing significantly caudally before a new tooth is added at the back, unlike in other taxa, such as captorhinids, where successive teeth are tightly packed together (de Ricqlès & Bolt, 1983; LeBlanc & Reisz, 2015). However, it could also relate to the lack of tooth replacement, which means that there are fewer generations of jawbone resorption and alveolar bone formation at each tooth position than there would be in a polyphyodont animal.

The ankylosed condition of the rhynchosaur tooth roots is clear: they are coated with both acellular and cellular cementum layers, with the latter completely contacting the alveolar bone around the root, leaving no intervening space (Figures 2, 3, 4 and 7, 8, 9). Although we did not directly observe gomphosis in our sample, Sharpey’s fibers anchoring the cellular cementum and alveolar bone layers (Figures 2b and 7b) confirm the presence of a periodontal ligament prior to ankylosis; Sharpey’s fibers would not be able to form within solid bone and would require an initial fibrous connection between tooth and bone. The lack of teeth preserved in the gomphosis stage in any of the rhynchosaur specimens we sampled may be the result of either a faster or earlier onset of ankylosis. As in some early synapsids (LeBlanc et al., 2018), the ligamentous phase of tooth attachment would have been brief, and the periodontal ligament would soon become enclosed by the centripetal growth of alveolar bone toward the cellular cementum. We interpret ankylosis as resulting from the centripetal growth of alveolar bone toward the cementum, rather than the reverse, as seen in mosasaurs (Caldwell et al., 2003), because the alveolar bone is thicker and more abundant than cellular cementum, even in newly ankylosed teeth: as shown in Figure 3d, the cementum is very thin, whereas the surrounding vascularized tissue appears to consist entirely of alveolar bone—similar to what is observed in silesaurids (Mestriner et al., 2022) and some early‐diverging synapsids (LeBlanc et al., 2018). This process of faster/earlier ankylosis likely explains why gomphosis is not observed in the histological sections, as well as why shed rhynchosaur teeth are never found isolated in the many Triassic fossil assemblages where remains of these animals are common. Once ankylosed, the teeth remained functional until their surfaces were worn flush against the bone surface (Figure 5a2), rather than being shed and replaced. This loss of replacement is accompanied by progressive structural changes; older teeth at the front of the jaw appear to have more compact (less spongy) alveolar bone and narrower pulp cavities compared to younger teeth at the back, which exhibit larger, open pulp cavities and spongier alveolar bone (Figures 1a,b, 5c, and 6). We interpret this as an adaptation to protect blood vessels from abrasion and impact in a region with a rich blood supply, strengthening the area and reducing its exposure to injury and infection. Over time, the newer teeth at the back would gradually begin to remodel and compact the alveolar bone and associated vasculature, also accumulating jawbone between adjacent teeth (as in Figure 9). Additionally, it is worth mentioning that, based on our observations, the areas between the teeth in Figure 5a1 have a smooth and shiny appearance, which may result from this remodeling process.

Our data add to a growing body of evidence that heterochronic changes in the timing and extent of mineralization led to the independent evolution of permanent gomphosis across many amniote lineages (LeBlanc et al., 2018; Mestriner et al., 2022). This includes archosauromorphs, in which differences between “ankylothecodonty” and thecodonty are related to the relative duration of the ligamentous phase (gomphosis) before the soft tissue mineralizes and fuses the tooth to the jaw (LeBlanc et al., 2018; Mestriner et al., 2022). In this context, our results indicate that rhynchosaurs exhibited a fast/early ankylosis (the “rapid ankylosis” of LeBlanc et al., 2018), similar to that of early‐diverging synapsids, where the ligamentous phase is brief, and the soft tissues quickly mineralize following tooth eruption. Accordingly, the early‐diverging phylogenetic position of rhynchosaurs within Archosauromorpha (Ezcurra et al., 2020) suggests that faster/earlier ankylosis likely represents the ancestral condition for the group. The later acquisitions of both “delayed ankylosis” and subsequent “permanent gomphosis” reflect heterochronic changes to the timing of mineralization, caused by a delay in the ankylosis process or and a truncation of the ligamentous phase (LeBlanc et al., 2018; Mestriner et al., 2022). Further investigation into the tooth attachment patterns of other early archosauromorphs is key to more comprehensively resolving the ancestral condition for the group, and the evolutionary transformations in tooth attachment tissue mineralization that would give rise to crocodylian and dinosaur gomphoses.

Lastly, the presence of plicidentine in our rhynchosaur sample represents the first discovery of this feature in archosauromorph teeth. This suggests that plicidentine, otherwise commonly found in sarcopterygians and early‐diverging tetrapods (Maho & Reisz, 2022; Maxwell, Caldwell, & Lamoureux, 2011c; Maxwell, Caldwell, Lamoureux, & Budney, 2011; Warren & Turner, 2006), might have persisted in many amniote lineages rather than being lost, as supported by its recent discovery in other groups, such as early eureptiles, parareptiles, synapsids, ichthyosaurs, choristoderes, varanoid lizards, and snakes (Brink et al., 2014; Kearney & Rieppel, 2006; MacDougall et al., 2014; Maxwell, Caldwell, & Lamoureux, 2011c; Maxwell, Caldwell, Lamoureux, & Budney, 2011; Palci et al., 2021).

### 5.2 Tooth emplacement and tooth attachment tissue formation

Most non‐avian archosauromorphs display continuous tooth replacement, but rhynchosaurs appear to have decoupled the formation of new teeth from the shedding of old ones (Benton, 1984; Sethapanichsakul et al., 2023). This spatial decoupling leads to the formation of multiple tooth rows (Figures 1a, 3a,b), similar to those found in captorhinids (de Ricqlès & Bolt, 1983; LeBlanc & Reisz, 2015), *Delorhynchus* (Rowe et al., 2021), and some anomodont synapsids (Olroyd et al., 2021). For this reason, we refer to the addition of new teeth to each row as tooth emplacement, rather than conventional tooth replacement (sensu LeBlanc & Reisz, 2015).

According to Benton (1984), as rhynchosaur jaws grew, rostral teeth were worn down and new teeth formed at the caudal and lingual margins of the jaws via the dental lamina (e.g., figure 5 of Sethapanichsakul et al. 2023). Hence, newly formed caudal teeth are larger than their rostral predecessors (Figures 1a, 5a,b, and 6) and the number of longitudinal tooth rows also increases with age (Sethapanichsakul et al., 2023). As a result, the caudal portions of the dentary and maxilla are labiolingually wider and have more tooth rows (Figures 1a, 4a, and 5a), reflecting overall body growth and the continuous caudal/lingual addition of teeth (Benton, 1984; Sethapanichsakul et al., 2023). Some lingual teeth have a close contact, occasionally resembling replacement events, where teeth appear to resorb one another (Figures 1f, 4b, and 5d,e). However, in this case, a new row appears to be in the process of being added, and we have also observed other teeth in close contact, in other parts of the jaw, without necessarily resorbing one another (Figure 6). There is a chunk of a remnant of dentine in NMT RB1627 that resembles a replacement event (“dr” in Figure 4b), and we do not completely discard the possibility of occasional replacements that occurred as the jaws were growing and incorporating new teeth. However, our results show that the vast majority of rhynchosaur teeth were not being replaced, supporting the findings from prior studies.

Although we did not directly observe gomphosis in any of the sampled rhynchosaur teeth, the presence of Sharpey’s fibers within the alveolar bone surrounding each tooth (Figures 2b and 7b) indicates that, after erupting into the oral cavity, new teeth were initially attached to the alveolar bone and cellular cementum via a periodontal ligament (LeBlanc et al., 2018; Mestriner et al., 2022). Indeed, we identified a recently emplaced tooth position along the caudolingual surface of the maxilla (NMT RB95) that preserves a tooth in the process of fusing to the jaw (“et” in Figure 3; Figure S1A). Thin sections of this tooth demonstrate the presence of multiple mineralized tissues forming the attachment system: distinct layers of acellular and cellular cementum coating the tooth root, which are fully formed, and a surrounding mass of thin alveolar bone trabeculae bridging the gap between the developing tooth, the neighboring tooth, and the jawbone (Figure 3c,d). The presence of three distinct mineralized tissues forming the periodontium in developing rhynchosaur teeth is untenable under the historic “bone of attachment” paradigm because this predicts that only a single type of mineralized tissue should exist in reptiles with ankylosed teeth (Tomes, 1882).

## 6 CONCLUSION

Histological analysis of *Stenaulorhynchus stockleyi* specimens from the Middle Triassic Manda Beds of Tanzania reveals that rhynchosaur “ankylothecodonty” involves cellular cementum, alveolar bone, and a (mineralized) periodontal ligament — tissues that are plesiomorphic to Amniota and seen in mammals, crocodylians, and dinosaurs. What was formerly described as “bone of attachment” is actually a misinterpretation of these three separate tissues, and rhynchosaur ankylosis occurred through fast/early mineralization of the periodontal ligament via incremental growth of alveolar bone after tooth eruption. This results in a short, transient period of gomphosis, difficult to detect without a more comprehensive sample. This condition, termed “rapid ankylosis”, is likely ancestral to Archosauromorpha, but additional data on tooth attachment of other early archosauromorphs is needed to confirm this hypothesis. Our findings further confirm earlier suggestions that rhynchosaur tooth rows increased in number as the animal aged, with new teeth added caudally and lingually without replacing existing ones, which were worn until non‐functional rather than being shed. Finally, rhynchosaur teeth were rooted in deep and persistent sockets composed of alveolar bone, consistent with the thecodont condition seen in other archosauromorphs.

## Supporting information


Data S1.

## Data Availability

The data that support the findings of this study are openly available in Zenodo at https://zenodo.org/uploads/15565455, reference number https://doi.org/10.5281/zenodo.15565455.
