# Supplementary material for: The histology of rhynchosaur (Diapsida, Archosauromorpha) ankylothecodonty
Source: J Anat. 2025 Aug 27;248(3):526–40. doi: 10.1111/joa.70037 (PMC12881868; doi:10.1111/joa.70037)
Supplement: Supplementary file 1 — Data S1. [file JOA-248-526-s001.docx]

**SUPPLEMENTARY ONLINE MATERIAL FOR**

The histology of rhynchosaur (Diapsida, Archosauromorpha) ankylothecodonty

Gabriel Mestriner^1,2,3^, Gregory F. Funston^3,4,5^, Sterling J. Nesbitt^6^, Júlio C. A. Marsola^7^, David C. Evans^2,3^, Christian A. Sidor^8^, Max C. Langer^1^, Aaron R.H. LeBlanc^9^

^1^Departamento de Biologia, Universidade de São Paulo, Avenida Bandeirantes 3900, Ribeirão Preto 14040-190, Brazil.

^2^Department of Ecology and Evolutionary Biology, University of Toronto, 25 Willcocks Street, Toronto, ON M5S 3B2, Canada.

^3^Department of Natural History, Royal Ontario Museum, 100 Queens Park Rd, Toronto, M5S 2C6 Ontario, Canada

^4^Department of Earth and Planetary Sciences, University of California Davis, 1 Shields Drive, Davis, California, USA

^5^Department of Anatomical Sciences, Renaissance School of Medicine, Stony Brook University, Health Sciences Center, 101 Nicolls Road, Stony Brook, New York 11794, USA.

^6^Department of Geosciences, Virginia Tech, Derring Hall 926 West Campus Drive, Blacksburg, VA 24061, U.S.A.

^7^Universidade Tecnológica Federal do Paraná, Dois Vizinhos, 85660-000, Estrada para Boa Esperança, km 04, Paraná, Brazil.

^8^Department of Biology and Burke Museum, University of Washington, Seattle, WA 98195.

^9^Faculty of Dentistry, Oral & Craniofacial Sciences 17th Floor Tower Wing, Guy’s Campus King’s College London London, UK.

**Data Availability Statement**: Raw histological data and CT scan files are available at: https://zenodo.org/uploads/15565455. For additional information or access to data not included in the repository, please contact the authors.


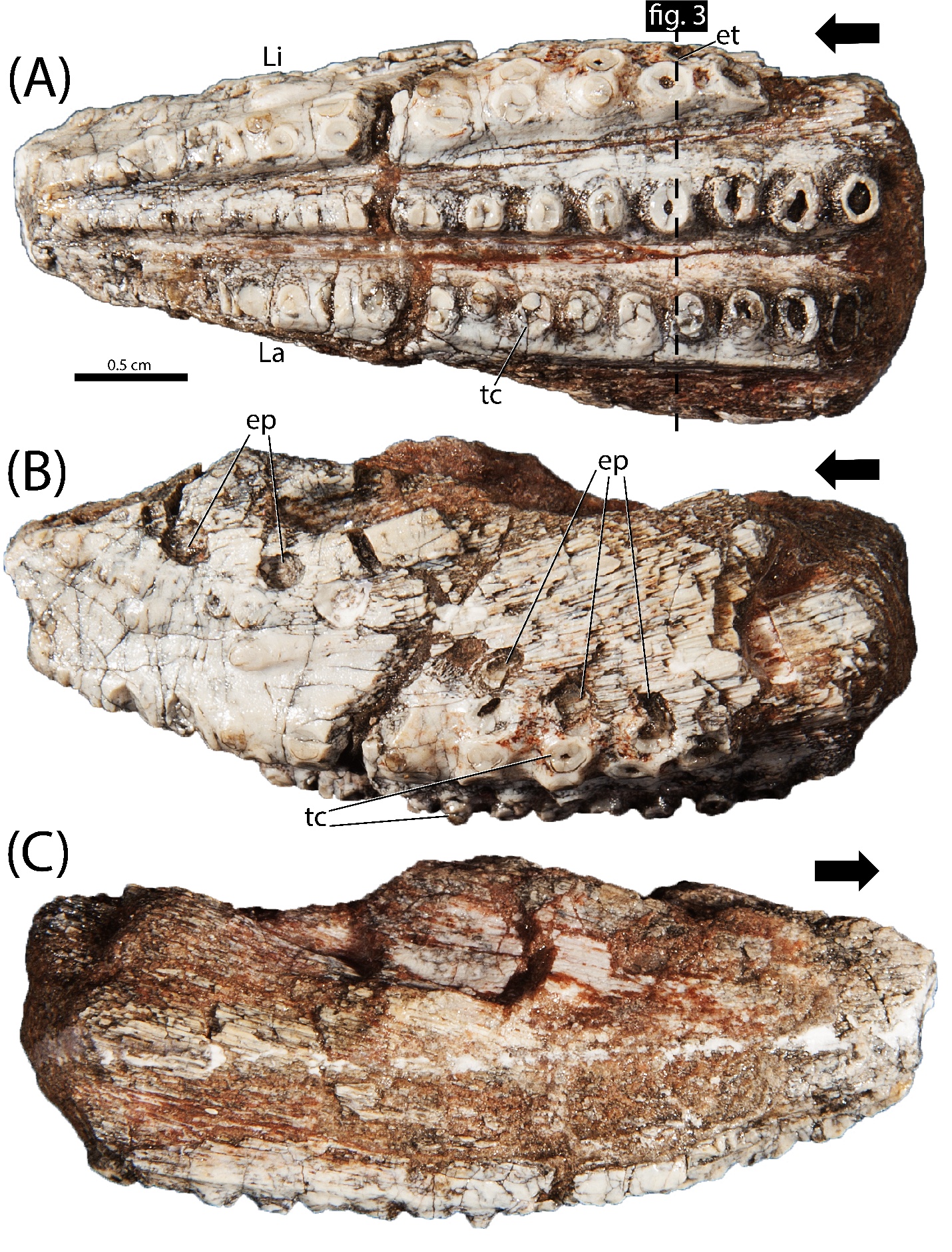
Supplementary figure 1. Right maxilla of *Stenaulorhynchus stockleyi* (NMT RB95) before thin sectioning, shown in ventral (A), lingual (B), and labial (C) views. Abbreviations: ep=emplacement pit; et=emplacement tooth; La=labial; Li=lingual; tc=tooth crown. Dashed line indicates the area where the coronal section was taken in Figure 3, showing the position of the emplacement tooth. Arrows indicate anterior direction.


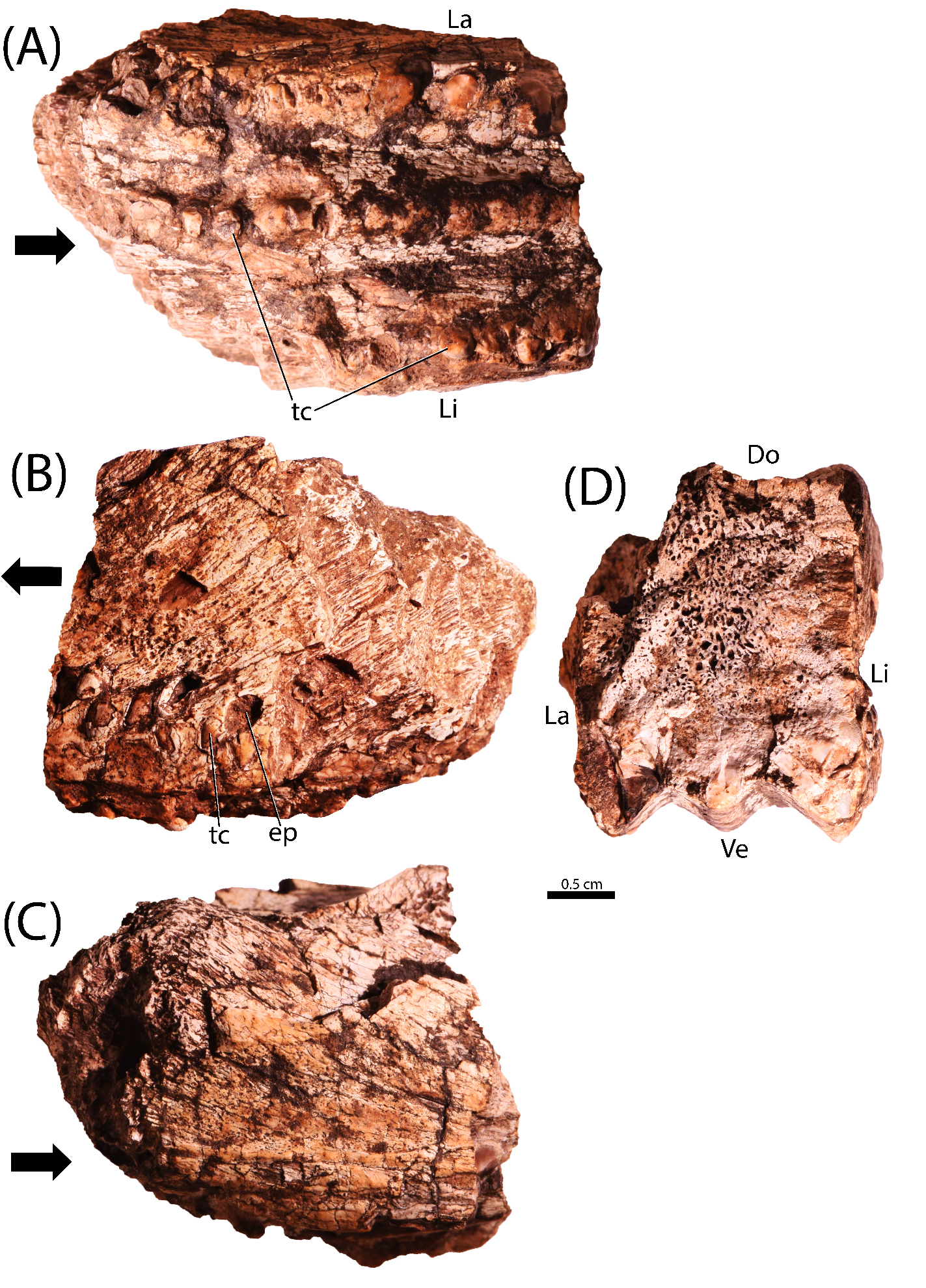
Supplementary figure 2. Right maxilla of *Stenaulorhynchus stockleyi* (NMT RB1627) before thin sectioning, shown in ventral (A), lingual (B), labial (C), and rostral (D) views. Abbreviations: ep=emplacement pit; do=dorsal; La=Labial; Li=Lingual; tc=tooth crown; ve=ventral. Arrows indicate anterior direction.


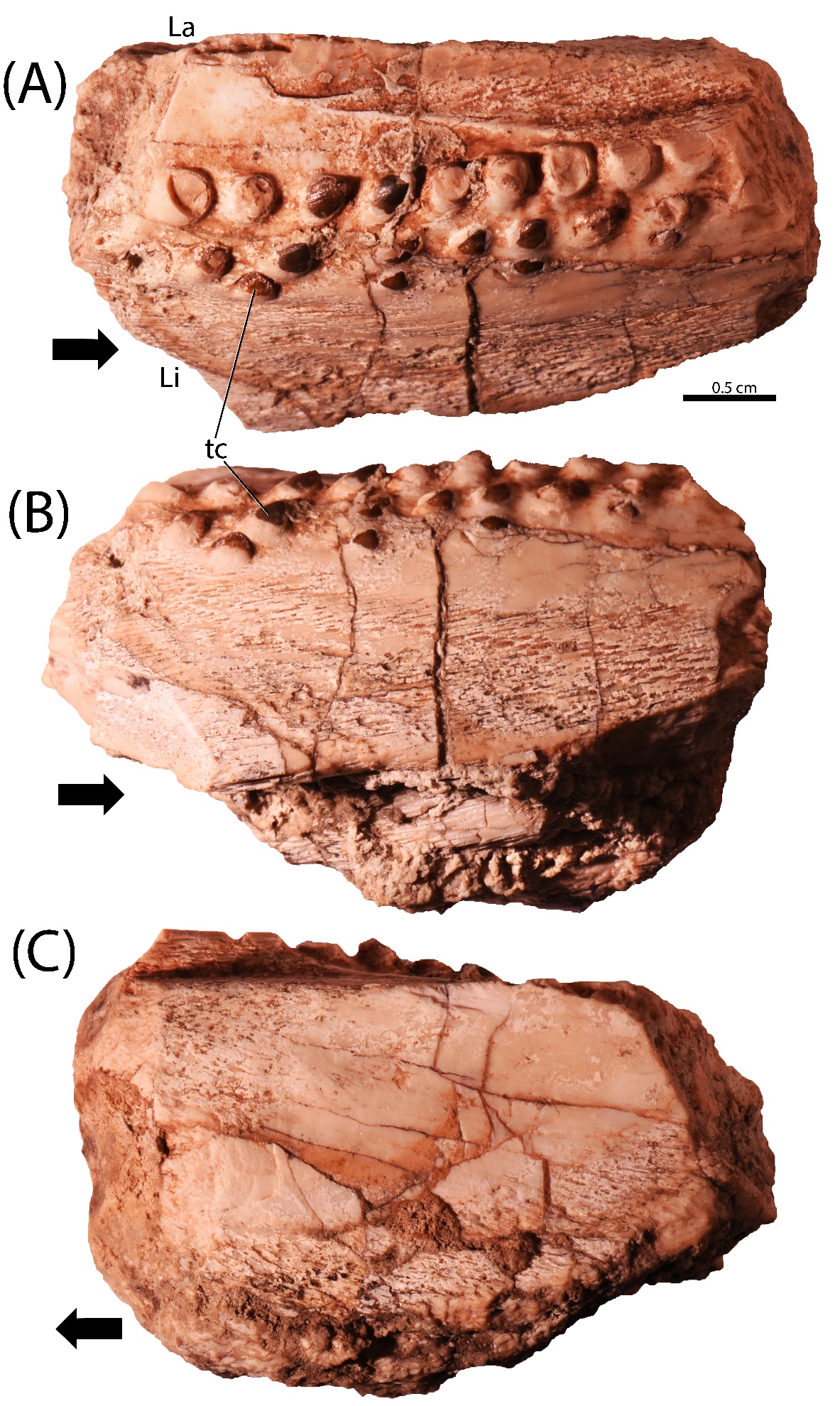


Supplementary figure 3. Left dentary of *Stenaulorhynchus stockleyi* (NMT RB1628) before thin sectioning, shown in dorsal (A), lingual (B), and labial (C) views. Abbreviations: La=Labial; Li=Lingual; tc=tooth crown. Arrows indicate anterior direction.
